# An emerging role of pendrin in health and disease

**DOI:** 10.14814/phy2.12503

**Published:** 2015-08-19

**Authors:** Yusuke Kumai, Dominique Eladari

**Affiliations:** 1 INSERM UMR_S 970, Equipe 12, Paris cardiovascular research center (PARCC)Paris, France; 2 Department of Physiology, Hopital Européen Georges Pompidou, AP-HPParis, France; 3 Faculté de Médecine Paris Descartes, Université Paris DescartesParis, France

The pendrin gene (*SLC26A4*) was initially identified through the positional cloning in patients with Pendred syndrome, a heritable recessive genetic disorder (MIM #274600) characterized by deafness and goiter (Everett et al. 1997). Pendrin encodes for a transmembrane electroneutral exchanger for monovalent anions (e.g. I^−^, formate, HCO_3_^−^, and Cl^−^). As predicted from the linkage between pendrin gene and characteristics of Pendred’s syndrome, pendrin is highly expressed in the inner ear and thyroid gland, where it controls the volume and pH of the endolymphatic fluid, and the transport of iodide required for organification of thyroid hormones, respectively. Pendrin is also highly expressed in the kidney where it controls the final excretion of bicarbonate (Royaux et al. 2001) and NaCl reabsorption and overall blood pressure (Eladari et al. 2014).

Although physiological roles of pendrin have been most extensively investigated in the three organs discussed above, pendrin is also expressed in airway epithelia, mammary gland and liver (Wangemann 2013). In these different organs, the function of pendrin is still unknown. However, there is an emerging interest in the relevance of pendrin in airway surface epithelia, because several studies show a possible association of pendrin with asthma. For example, Nakao et al. (Nakao et al. 2008) demonstrated that forced expression of pendrin in human carcinogenic cell line (NCI-H292) and mouse lung significantly increased expression of mucin (Muc5ac), a component of the mucus. In addition to these changes, pendrin overexpression led to elevated airway hyper-reactivity. Furthermore, Nakagami et al. (Nakagami et al. 2008) also showed that although there was no significant difference under the baseline condition between wild-type and pendrin knockout mice, inflammation and airway hyper-reactivity induced by ovalbumin injection were significantly alleviated in pendrin knockout mice.

While the results from mouse models suggest that abnormal overexpression of pendrin in airway epithelia could lead to severe airway inflammation, for clinical purposes it is imperative to test the role of pendrin in airway epithelia directly using human tissue. To address this point, the present study by Lee et al. (Lee et al. in press[Bibr b4]) utilized primary cell culture established from human nasal epithelial cells collected from either human subjects harboring bi-allelic inactivating mutations in pendrin or subjects with functional pendrin proteins. They treated these two primary cultures with a T_H_2-type cytokine (IL-13), one of the key mediators in inflammatory response in asthma, and analyzed the ASL thickness, expression and activities of selected ion transporters expressed on human nasal epithelia, as well as status of goblet cells, a specialized mucus-secreting cells.

Because nasal epithelia express a variety of transporters, one important question is to determine the relative role of pendrin in electrolyte transport in these cells. At mRNA level, Lee et al. detected 9 anion exchangers in addition to pendrin. Among those genes, only SLC26A3 was significantly elevated in response to IL-13 treatment, and the magnitude of increase was much lower than that of pendrin (roughly 10 and 100-fold increase for SLC26A3 and pendrin, respectively). Furthermore, following the IL-13 treatment, no increase in Cl^−^-dependent HCO_3_^−^ transport activity was observed in the pendrin-null cell culture. In addition to the anion exchangers, they also compared the mRNA expression and activity level of epithelial Na^+^ channel (ENaC), ANO-1 (Ca^2+^-dependent Cl^−^ channel) and cystic fibrosis transmembrane conductance regulator (CFTR) between the two cultures under the baseline condition. The only significant difference observed in this study was reduction in mRNA expression of CFTR in the pendrin-null cell culture. Overall, pendrin is likely to be the major mediator of Cl^−^-HCO_3_^−^ exchange activity in nasal epithelia following IL-13 treatment, but at the same time, the loss of pendrin had very little effect on the expression of other transporters.

On the other hand, the loss of pendrin led to major physiological changes in nasal epithelia. Unlike results from mouse models, the ASL was significantly thicker in pendrin-null cell culture even in the baseline condition, and the IL-13 treatment significantly increased the ASL thickness in both types of cell cultures. In addition to abnormal ASL thickness, in the pendrin-null cell culture, the IL-13 treatment did not stimulate the expression of Muc5ac, or induce hyperplasia in goblet cells as visualized by Periodic-acid Schaff stain.

Taken together, results of Lee et al. show that previous results obtained from mouse models are largely applicable to human nasal epithelia. That is, the loss of pendrin reduces mucus secretion and increases the ASL thickness at the same time, and likely to alleviate the hyperreactivity of lung airway. This makes pendrin a potential therapeutic target for asthma. However, several questions remain to be addressed. For example, what would be the role of pendrin in controlling ASL under basal condition, especially in comparison to other ion transporters expressed on the epithelia? Determining if the loss of pendrin has an effect on ASL in vivo, as well as in vitro, in the human airway would be relevant to understand the ASL homeostasis regulation. Furthermore, there still is a very simple mechanistic question—“how exactly does the inhibition of pendrin affect the ASL thickness?” Because the ASL thickness is controlled by the solute transport and ASL osmolarity, one possibility is that loss of pendrin is simply affecting overall osmolarity of the ASL, as described in the schematics presented by the authors of the article. An alternative, although speculative, possibility is that the loss of pendrin alters pH of the ASL (Garnett et al. 2011), and subsequently affects the relative density of mucus-secreting goblet cells and water (and solute) secreting serous cells in the nasal epithelia. In this case, the linkage between the loss of pendrin and the increased ASL would be primarily due to altered cell differentiation, rather than just simple changes in the overall salt transport (and ASL osmolarity).

In conclusion, Lee et al. provides additional support for the linkage between pendrin and lung epithelial pathophysiology. Future studies should emphasize on clarifying the molecular basis for the linkage between pendrin function and lung epithelial physiology. Insights from such studies would be invaluable to solidly establish pendrin as a therapeutic target of asthma.
